# Local structures of mesoporous bioactive glasses and their surface alterations *in vitro*: inferences from solid-state nuclear magnetic resonance

**DOI:** 10.1098/rsta.2011.0257

**Published:** 2012-03-28

**Authors:** Philips N. Gunawidjaja, Renny Mathew, Andy Y. H. Lo, Isabel Izquierdo-Barba, Ana García, Daniel Arcos, María Vallet-Regí Mattias Edén

**Affiliations:** 1 Physical Chemistry Division, Department of Materials and Environmental Chemistry, Arrhenius Laboratory, Stockholm University, 106 91 Stockholm, Sweden; 2 Departamento de Química Inorgánica y Bioinorgánica, Facultad de Farmacia, Universidad Complutense de Madrid, 28040 Madrid, Spain; 3 Networking Research Center on Bioengineering, Biomaterials and Nanomedicine (CIBER-BBN), Madrid, Spain

**Keywords:** silicate-based biomaterial, biomineralization, magic angle spinning ^29^Si NMR, cross polarization, surface reactions

## Abstract

We review the benefits of using ^29^Si and ^1^H magic angle spinning (MAS) nuclear magnetic resonance (NMR) spectroscopy for probing the local structures of both bulk and surface portions of mesoporous bioactive glasses (MBGs) of the CaO–SiO_2_−(P_2_O_5_) system. These mesoporous materials exhibit an ordered pore arrangement, and are promising candidates for improved bone and tooth implants. We discuss experimental MAS NMR results from three MBGs displaying different Ca, Si and P contents: the ^29^Si NMR spectra were recorded either directly by employing radio-frequency pulses to ^29^Si, or by magnetization transfers from neighbouring protons using cross polarization, thereby providing quantitative information about the silicate speciation present in the pore wall and at the MBG surface, respectively. The surface modifications were monitored for the three MBGs during their immersion in a simulated body fluid (SBF) for intervals between 30 min and one week. The results were formulated as a reaction sequence describing the interconversions between the distinct silicate species. We generally observed a depletion of Ca^2+^ ions at the MBG surface, and a minor condensation of the silicate-surface network over one week of SBF soaking.

## 1. Introduction

Silica-based *melt-prepared bioactive glasses* (MPBGs) [1–4] are in clinical use for repairing fractures and filling voids in bone and tooth. When subjected to body fluids, they bond chemically to human tissues by the precipitation of an initially amorphous calcium phosphate (ACP) layer that later transforms into Ca-deficient nanocrystalline hydroxy-carbonate apatite (HCA); the latter closely resembles the inorganic constituent of biomineralized bone and tooth [5].

**Figure 1.**
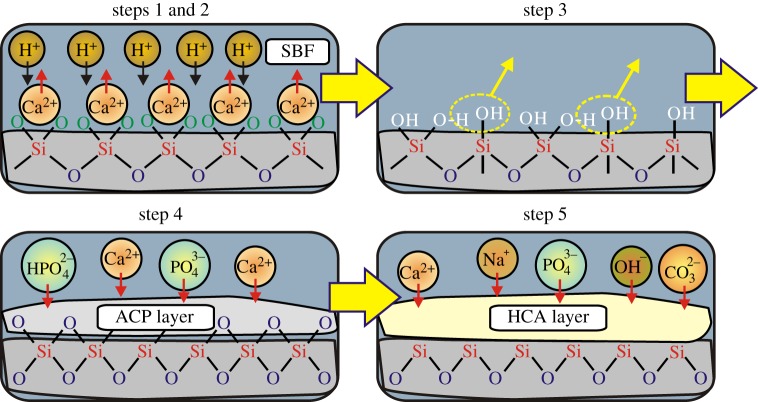
Schematic illustration of the reaction sequence leading to HCA formation according to Hench and co-workers [1,6], here assuming a melt-prepared CaO–SiO_2_ glass. The first three stages involve reactions between the silicate surface and the surrounding fluid as follows: (1) Ca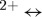 H^+^ exchange and (2) breakage of Si–O–Si bonds, leading to the formation of Si–OH groups; (3) repolymerization: 2Si–OH→Si–O–Si+H_2_O. Next follows (4) the formation of amorphous calcium phosphate (ACP) and (5) ACP→HCA crystallization that involves uptake of additional ions, e.g. OH^−^, 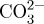 and Na^+^. (Online version in colour.)

Hench and co-workers suggested a reaction sequence comprising five main steps that account for the formation of HCA at the MPBG surface on its exposure either to body fluids *in vivo* or to a simulated body fluid (SBF) *in vitro* [1,6]. This gross scheme is outlined in figure 1 and will henceforth be referred to as the *Hench mechanism* (HM). Its first three steps involve reactions at the silicate surface (that will be addressed herein), whereas stages 4 and 5 imply formation of ACP and HCA, respectively. A large number of experimental [1,2,6–14] and numerical [15–19] studies overall validate the HM: yet, many of its details are poorly understood, particularly the optimal silicate surface characteristics and the elementary reactions that initiate ACP formation, as well as details of the ACP→HCA crystallization.

The precise molecular mechanism underlying the HM presumably varies slightly depending on the structural, textural and compositional properties of the biomaterial [14] and the exact external conditions of its surrounding medium. Such issues are important to settle in view of the currently rapid developments of improved bioactive glasses (BGs), notably those prepared by using structure-directing agents, e.g. *mesoporous bioactive glasses* (MBGs) of the CaO–SiO_2_−P_2_O_5_ system [13,20–24]. Thanks to their large surface area, *ordered*arrangement of mesopores and unique pore-wall structure (see §3), they exhibit higher bioactivity (i.e. faster HCA formation) than MPBGs.

This contribution serves a dual purpose: on the one hand, it reviews the advantages of using ^29^Si and ^1^H solid-state nuclear magnetic resonance (NMR) for probing both the bulk and surface of MBGs. We also discuss our recently proposed structural model of the MBG pore wall [23]. On the other hand, this article presents new experimental data monitoring the surface reactions observed from MBGs comprising different Ca, Si and P contents; they are contrasted and discussed in relation to the three first steps of the HM.

This paper is organized as follows: §2 provides experimental details and introduces the sample notation employed for our three series of SBF-exposed MBGs. Section 3 reviews general aspects of the MBG structure, and illustrates how magic angle spinning (MAS) NMR may be exploited to investigate them. Section 4 presents and discusses the ^29^Si MAS NMR probing of the MBG structures and their surface reactions occurring during one week of SBF immersion, whereas §5 relates them to the HM. Section 6 accounts for the various proton species observed at the MBG surface by ^1^H NMR, and §7 summarizes our main findings.

## 2. Material and methods

### (a) Sample preparations

The MBG synthesis involved an evaporation-induced self-assembly process [25] at 40^°^C, using the P123 triblock copolymer as the structure-directing agent, as described by López-Noriega *et al*. [22]. Each of the elements Si, P and Ca were incorporated using precursors of tetraethyl orthosilicate, triethyl phosphate and Ca(NO_3_)_2_⋅4H_2_O, respectively. The resulting homogeneous membranes were heated at 700^°^C for 6 h to remove organic species and nitrate ions. This procedure was employed to prepare three MBG specimens of nominal molar compositions 10CaO–90SiO_2_, 10CaO–85SiO_2_−5P_2_O_5_ and 37CaO–58SiO_2_−5P_2_O_5_, labelled ‘S90’, ‘S85’ and ‘S58’, respectively, according to their mol% of SiO_2_.

Analysed cation compositions were determined by X-ray fluorescence (XRF) spectroscopy using a Philips PANalytical AXIOS spectrometer (Philips Electronics NV) with X-rays generated by the Rh *K*_α_ line at *λ*=0.614 Å. Overall, very good agreement resulted between the batched and the analysed compositions, in which the largest relative deviations were observed for phosphorus (table 1).

**Table 1.** Nominal and analysed MBG compositions.

sample	oxide equivalent^a^	nominal composition^a^	analysed composition^b^	split representation^c^
S90	10CaO–90SiO_2_	Ca_0.111_SiO_2.11_	Ca_0.101_SiO_2.10_	Ca_0.101_SiO_2.10_
S85	10CaO–85SiO_2_−5*P*_2_O_5_	Ca_0.118_SiP_0.118_O_2.41_	Ca_0.125_SiP_0.051_O_2.25_	[Ca_0.049_SiO_2.05_]
				−0.051[Ca_3/2_PO_4_]
S58	37CaO–58SiO_2_−5*P*_2_O_5_	Ca_0.638_SiP_0.172_O_3.07_	Ca_0.659_SiP_0.148_O_3.03_	[Ca_0.437_SiO_2.44_]
				−0.148[Ca_3/2_PO_4_]

^a^Nominal batched composition, expressed as molar oxide equivalents, or using a stoichiometric formula normalized to a unity Si coefficient.

^b^Calculated from the XRF-analysed cation composition, with the oxygen coefficient obtained through charge balance.

^c^Composition according to equation (3.1).

### (b) In vitro *studies*

An SBF solution was prepared according to Kokubo *et al*. [26] by dissolving NaCl, KCl, NaHCO_3_, K_2_HPO_4_⋅3H_2_O, MgCl_2_⋅6H_2_O, CaCl_2_ and Na_2_SO_4_ in distilled water. It was buffered at pH = 7.45 by using tris(hydroxymethyl)–aminomethane/HCl and subsequently passed through 0.22 μm Millipore filters to avoid bacterial contamination. A 1.00 g sample of each pristine MBG (in the form of grains of variable sizes from a few micrometres to approx. 0.5 mm in diameter) was immersed in 50 ml of SBF under continuous orbital stirring (100 r.p.m.) for variable intervals between 0.5 h and 7 days. The sealed polyethylene containers were placed in an Ecotron HT incubator at 37^°^C. Each sample was filtered, washed with water to quench the surface reactions and subsequently vacuum dried at 37^°^C for several days.

The resulting SBF-soaked specimens are denoted as S90-*τ*_SBF_, S85-*τ*_SBF_ and S58-*τ*_SBF_, with the immersion period *τ*_SBF_ specified either in hours (h) or days (d).

### (c) Solid-state nuclear magnetic resonance

The NMR experimentation was performed on finely ground powders of the S90, S85 and S58 derived samples, by filling 6 mm zirconia pencil rotors and undergoing MAS at a rate of *ω*_*r*_/2*π*=8.0 kHz for all ^29^Si acquisitions. An Agilent/Varian/Chemagnetics Infinity-400 spectrometer was employed at a magnetic field of 9.4 T, giving Larmor frequencies of 79.5 MHz for ^29^Si and −400.1 MHz for ^1^H. All ^29^Si NMR data for S85 were previously presented in Gunawidjaja *et al*. [14].

Single-pulse ^29^Si NMR experiments employed a nutation frequency of *ω*^Si^_nut_/2*π*=37 kHz, with the flip angle and relaxation delay as follows: 70^°^ and 720–1080 s, respectively, for all samples associated with S90 and S85; 60^°^ and 1800 s for those derived from S58. The relaxation delay was selected for each sample based on a separate spin–lattice relaxation (*T*_1_) measurement. Typically, 260–300 signal transients were co-added. ^1^H NMR spectra were recorded by Hahn spin–echoes [27] at *ω*_*r*_/2*π*=9.0 kHz using *ω*^H^_nut_/2*π*=48 kHz (also employed for the ^1^H 90^°^ pulse in all cross polarization (CP) acquisitions), echo delay *τ*_echo_=111 μs, 5.0 s relaxation delay and approximately 1000 transients/acquisition.

Ramped [28] ^1^H→^29^Si CP was established at the modified Hartmann–Hahn condition *ω*^H^_nut_−*ω*^Si^_nut_=*ω*_*r*_, giving nutation frequencies of 19 kHz and 27 kHz for ^29^Si and ^1^H, respectively. The number of accumulated transients was chosen depending on the Si content of the sample and varied as follows: 6000–16 000 for the S90 and S85 series and 25 000–30 700 for that of S58. The contact interval (*τ*_CP_) was 2.0 ms and the relaxation delay was 5.0 s throughout.

We verified that application of high-power ^1^H decoupling did not affect the ^29^Si NMR peak widths perceptibly, and all experimentation was performed without ^1^H decoupling. The processing involved 150 Hz Gaussian signal apodization. The MAS rate of 8.0 kHz is sufficiently fast to concentrate >95% of the integrated ^29^Si NMR signal intensity into the centre-band; hence, all NMR spectra presented below are zoomed around the centre-band region. Chemical shifts are quoted relative to neat tetramethylsilane.

Each ^29^Si NMR spectrum was deconvoluted into its underlying Gaussian signal components by employing an in-house iterative fitting computer program that allows restrictions to be imposed for each peak position and full width at half maximum (FWHM) height. With all peak positions bound within (at least) ±4 ppm and the FWHM restricted between 7 ppm and 13 ppm, each NMR spectrum was fitted several times (>10), using different initial conditions and shift boundaries. The resulting set of best-fit parameters was used to derive the mean values and standard deviations of the respective parameters. They varied marginally between the samples.

## 3. Mesoporous bioactive glasses and ^29^Si nuclear magnetic resonance: an overview

Here, we review structural features of the MBG pore wall over both atomic and nanometre length scales. Further, we briefly outline routine solid-state NMR experimentation targeting ^1^H and ^29^Si [29–31], and illustrate how it may be exploited to probe the SiO_4_ speciations at both the pore-wall surface and its interior, as well as monitoring the surface modifications following SBF soaking of the MBG materials.

### (a) Structural building blocks of mesoporous silica and silicate glasses

MBGs share structural features with both porous silica and traditional melt-prepared glasses of the CaO–SiO_2_−(P_2_O_5_) system, as discussed previously [14,23]. Their textural properties, such as a large specific surface area, are similar to those of silica-based micro- and mesoporous materials. The pore walls of mesoporous silica are built from a three-dimensional network of SiO_4_ units, interconnected by oxygen bridges at each corner of the tetrahedra. The structural building blocks of silicates are commonly described using the *Q*^*n*^ notation [30,31], where *n* denotes the number of bridging oxygen (BO) atoms at the SiO_4_ tetrahedron, leaving 4–*n* positions occupied by non-bridging oxygen (NBO) ions. Hence, the pore-wall interior of mesoporous silica is constructed predominantly by *Q*^4^ units.

**Figure 2.**
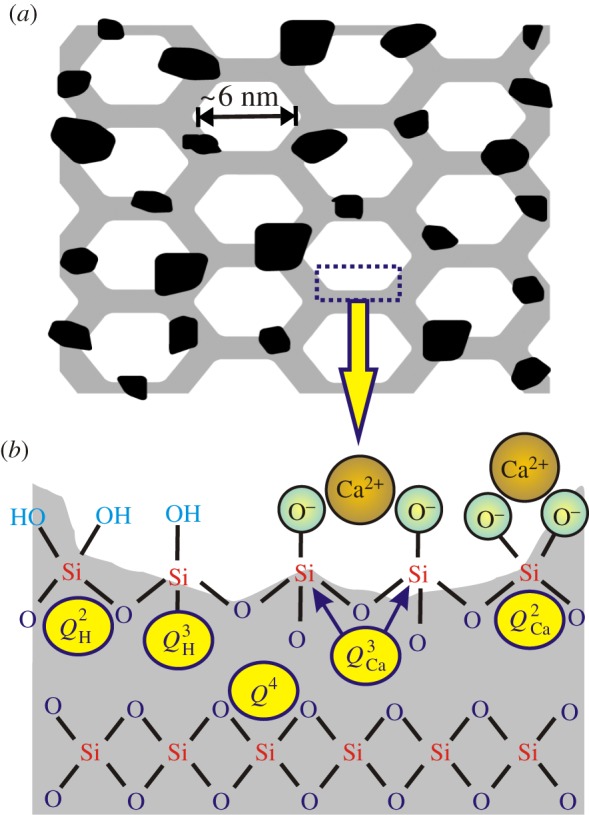
(*a*) Structural model of the MBG pore wall [23], consisting of a primary CaO–SiO_2_ phase (grey), with inclusions of nanometre-sized calcium orthophosphate (CaP) clusters (black). (*b*) Schematic picture of the interior and surface of the silica-based constituent. The pore-wall interior is primarily built by interconnected *Q*^4^ silicate tetrahedra, whereas the surface incorporates silanols comprising one (*Q*^3^_H_) and two (*Q*^2^_H_) hydroxyl groups (cyan), as well as Ca^2+^-associated *Q*^3^_Ca_ and *Q*^2^_Ca_ tetrahedral units. Marine blue and green oxygen atoms denote BO and NBO (encircled) species, respectively. (Online version in colour.)

It is well known that the *surface* of a mesoporous material is rich in silanols, i.e. lower connectivity SiO_3_(OH) and SiO_2_(OH)_2_ moieties involving terminal Si–OH groups; they will be denoted by 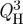 and *Q*^2^_H_, respectively, to stress that protons provide charge compensation of the NBOs [23]. Figure 2 provides a schematic picture of the various *Q*^*n*^ building blocks. Because of the distinct electronic configurations at their ^29^Si nuclei, these tetrahedral units are readily identified by ^29^Si NMR, as they produce signals at chemical shifts separated by about 10 ppm in the spectrum [29–35]. In the case of our MBGs, we observe the *Q*^4^, 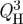 and *Q*^2^_H_ resonances around −110, −101 and −91 ppm, respectively.

MBGs also display structural characteristics of conventional melt-prepared glasses, as their *amorphous* pore walls constitute a silicate network, weakly modified by Ca^2+^ ions. The latter break Si–O–Si bonds, leading to the formation of *Q*^*n*^_Ca_ species (*n*=1, 2, 3), where each Ca^2+^ ion may (for instance) charge balance either the NBOs at *one Q*^2^_Ca_ tetrahedron (i.e. SiO_2_(O^−^)_2_) or *two* neighbouring *Q*^3^_Ca_units (i.e. SiO_3_O^−^), as illustrated in figure 2. If spectral resolution permits, ^29^Si NMR may identify such silicate units based on their distinct chemical shifts, which typically appear around −90 ppm (*Q*^3^_Ca_), −83 ppm (*Q*^2^_Ca_) and −75 ppm (*Q*^1^_Ca_) [30,31,36,37].

Yet, *if* all six members of the set of silicate species {*Q*^4^,*Q*^3^_H_,*Q*^2^_H_,*Q*^*n*^_Ca_} are simultaneously present in the structure, the ^29^Si NMR signal from each unit may usually not be unambiguously resolved. This stems from the structurally disordered nature of the pore walls, and the chemical shift of each ^29^SiO_4_ tetrahedron also depends on a multitude of local structural parameters, such as Si–O distances and Si–O–Si bond angles, as well as the precise location of the Ca^2+^ ions [30,31]. Since each such parameter of a given *Q*^*n*^ species is associated with a distribution across the material, its net ^29^Si NMR response becomes very broad, typically 7–12 ppm. Unfortunately, the signal *separation* between members within each group of {*Q*^*n*^_Ca_} and {*Q*^*n*^_H_} units is of similar size to their peak widths. Further, the *Q*^2^_H_ and *Q*^3^_Ca_ species exhibit almost identical mean chemical shifts.

### (b) Essential solid-state nuclear magnetic resonance experimentation

The quantification of the various *Q*^*n*^ silicate populations is normally performed by ‘directly’ exciting the NMR signal from the sample by applying a radio-frequency (rf) pulse prior to its recording (often referred to as ‘single-pulse’ or ‘Bloch-decay’ acquisition), followed by spectral deconvolution involving iterative fitting. ^1^H→^29^Si CP combined with MAS (CPMAS) is a widely employed NMR tool capable of providing *selective* information about the silicate surface speciation, stemming from an arrangement of the sole excitation of NMR responses from ^29^Si nuclei in close proximity to nearby protons [29–35]. Such selectivity follows naturally from the presence of large amounts of silanols and physisorbed water molecules at the MBG surface, whereas the proton abundance is very low in the pore-wall interior. By carefully selecting the so-called *contact interval* (*τ*_CP_)—during which rf fields are applied simultaneously to the ^1^H and ^29^Si nuclei—one may gain some control of the depth over which the ^29^Si are detected. Using short values of *τ*_CP_<0.5 ms restricts the probing of ^29^Si at the ^1^H-associated surface, whereas prolonged rf application (>10 ms) may affect ^1^H→^29^Si magnetization transfers beyond 0.5 nm. Provided that relatively short contact intervals (*τ*_CP_≤2 ms) are employed, CPMAS-acquired NMR spectra faithfully quantify the various *Q*^*n*^_H_ and *Q*^*n*^_Ca_ populations at the MBG surface, as illustrated by Gunawidjaja *et al*. [14].

We recently demonstrated the utility of ^1^H→^29^Si CPMAS for probing the S85 MBG surface and its alterations during prolonged SBF immersion [14,23]. These surface reactions will be elucidated further herein, where we compare NMR results from three MBG series of variable Ca, Si and P contents.

### (c) Constitution of phosphorus-bearing pore walls: calcium phosphate clusters

Phosphorus-bearing MBGs are reported to provide faster HCA formation than their CaO–SiO_2_ and pure silica counterparts [21,24], in agreement with general observations from studies of melt- and sol–gel-prepared BGs *in vitro* [1,2,12,38,39]. The presence of phosphorus in CaO–SiO_2_−P_2_O_5_ MBGs triggers questions regarding its structural role in the pore wall, primarily its relationship to Ca and Si. Although initial studies based on transmission electron microscopy coupled with energy-dispersive X-ray spectroscopy indicated a homogeneous element distribution over tens of nanometres [13,20–22], we recently clarified the role of P at the pore wall by employing complementary solid-state NMR experiments [23]: phosphorus is present exclusively as a *separate* calcium *ortho*phosphate ‘phase’. We suggested that it forms nanometre-sized *disordered* clusters (denoted ‘CaP’), interrupting the dominating CaO–SiO_2_ pore-wall builder [23]: a schematic picture is given in figure 2. A main consequence of this ‘bi-phasic’ pore-wall model is the inherently high accessibility of the CaP clusters to their surrounding medium, which naturally explains [14,23,24] the well-documented substantial and rapid (minutes to hours) release of Ca^2+^ and PO^3−^_4_ ions from MBGs into the SBF [13,14,20–22,40].

Despite the major fraction of the CaP clusters dissolving within the first few hours of MBG immersion, it is likely that some clusters remain intact at the pore wall, where they may act as nucleation sites for further growth into an ACP layer [14,23,24]. Nevertheless, ultimate proof for the existence of the amorphous CaP clusters is yet to be established and further studies are required to clarify their potential role in the HCA formation.

### (d) The CaO–SiO_2_ pore-wall component

*Regardless* of the existence of CaP clusters, the unambiguous sole presence of *ortho*phosphate ions in these MBG structures [14,23,24] restricts the amount of Ca^2+^ ions available for modifying the silica-based pore-wall portion: as the 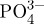 ions consume a significant fraction of the Ca^2+^ modifiers for maintaining charge balance, the extent of *Q*^4^→*Q*^*n*^_Ca_ (*n*=3, 2, 1) conversions reduces, i.e. the level of silicate network depolymerization [14,23].

While the exact composition of the CaP pore-wall constituent is unknown, and solid-state NMR confirms the presence of some apatite-like OH moieties [23,41], it is reasonable to assume a stoichiometric Ca_3_(PO_4_)_2_ composition. As we are for the moment interested only in the effects of Ca^2+^ on the silicate network and its associated *Q*^*n*^-speciation, the MBG compositions listed in table 1 ignore the proton content at the surface, i.e. the presence of 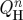 tetrahedra. Generally, the net stoichiometric MBG formula, Ca_*q*_SiP_*p*_O_2+*q*+5*p*/2_, may equivalently be cast as [14,23,42,43]
3.1
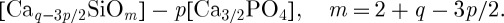
Equation (3.1) is a special case of the ‘split-network’ analysis discussed earlier [42,43], from which we may draw the following qualitative conclusions. (i) It accounts for the Ca^2+^ consumption by the *orthophosphate* ions, where the amount of Ca^2+^ ions associated with the silicate pore-wall component *reduces* for increasing P content of the MBG. The entire Ca reservoir is located in the phosphate phase when *q*≈3*p*/2. This argument remains qualitatively valid over the entire range of conceivable molar ratios between Ca and P (approx. 1.0–1.7) in the calcium phosphate pore-wall portion. (ii) The *average* polymerization degree of the *silicate* network is (approximately) given by *r*_*Si*_=*m*=2+*q*−3*p*/2, translating into the average number of BO atoms (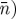 from the set {*Q*^4^, *Q*^*n*^_Ca_} of silicate tetrahedra being equal to [30,42–46]
3.2
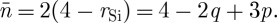


Using equation (3.1), we may express the analysed stoichiometric formula for each of the S58, S85 and S90 MBGs according to the ‘split representation’ given in table 1. For these MBG compositions, we conclude the following. (**A**) Despite the S90 and S85 specimens comprising nearly equal Ca contents (table 1), the absence of P in the S90 MBG should effect a higher degree of silicate network depolymerization, i.e. a larger relative population of *Q*^*n*^_Ca_ (*n*≤3) units than S85. (**B**) In the S85 structure, very few Ca^2+^ ions remain for creating significant amounts of *Q*^*n*^_Ca_ units. (**C**) The markedly larger Ca content of the S58 composition (while its molar fraction of P is only slightly higher than that of S85) predicts a significantly larger amount of Ca^2+^-associated SiO_4_ units when compared with the other MBG samples.

In §4, predictions (**A**)–(**C**) will be verified experimentally by single pulse-acquired ^29^Si NMR spectra that quantitatively reflect the *entire* silicate speciation, as well as by ^1^H→^29^Si CP that selectively probes the *surface*.

## 4. The silicate speciations of the S90, S85 and S58 mesoporous bioactive glasses

### (a) Pristine mesoporous bioactive glasses

#### (i) Single-pulse nuclear magnetic resonance: quantitative pore-wall ^29^Si speciations

**Figure 3.**
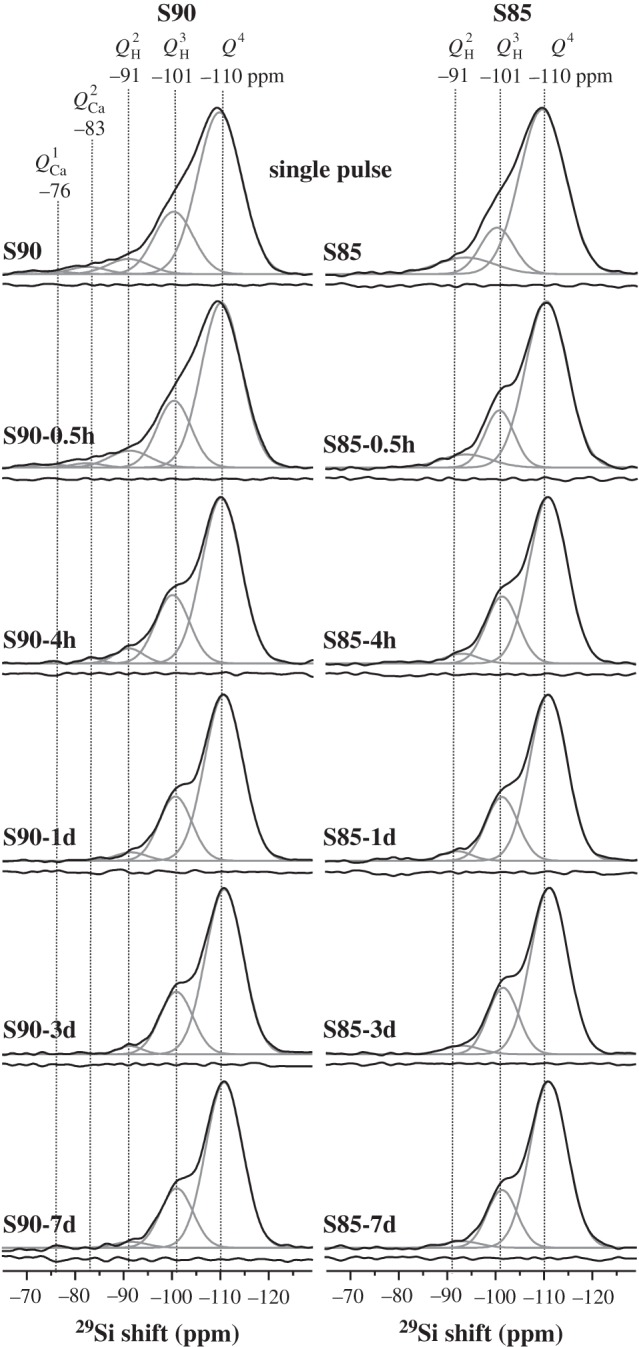
Directly excited ^29^Si MAS NMR spectra (black lines) from pristine S90 and S85 (top row). All other spectra were recorded after SBF immersion from the as-indicated MBG samples. Deconvoluted peak components are plotted using grey lines; their assignments are displayed at the top of each column. The curve beneath each spectrum represents the difference between the experiment and best fit.

**Figure 4.**
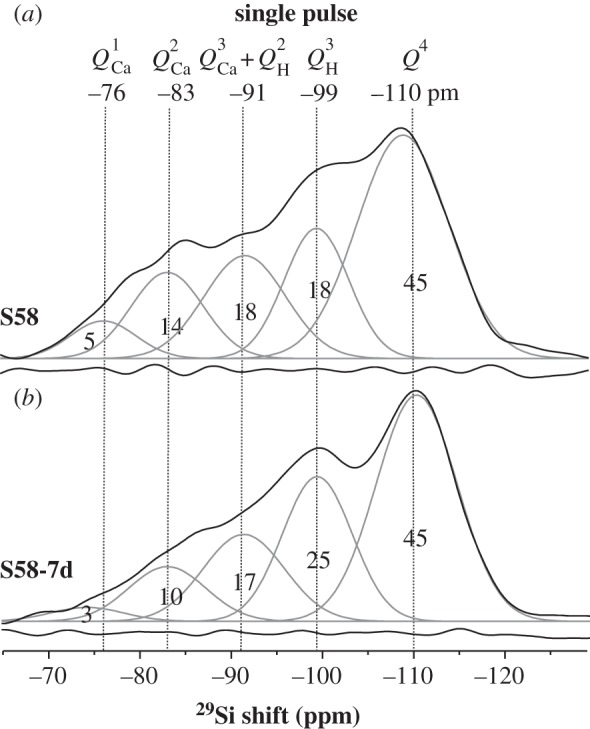
(*a*) Directly excited ^29^Si MAS NMR spectra from the pristine S58 sample and (*b*) after its immersion in SBF for one week. The number at each peak component represents its relative population (in per cent), and other labels are as in figure 3.

The directly excited ^29^Si NMR spectra from the S90 and S85 specimens are displayed in figure 3, together with deconvolutions into their underlying peak components. Owing to the very long experimental times required to achieve reasonable NMR spectral signal-to-noise ratios (S/N) from the S58 samples (stemming from a lower Si content, coupled with slower ^29^Si *T*_1_ relaxation), figure 4 only includes the two extreme members of this series, i.e. S58 and S58-7d. Best fit NMR parameters and fractional populations of the *Q*^4^, *Q*^*n*^_H_ and *Q*^*n*^_Ca_ species are collected for all samples as shown in table 2. We initially focus on the results from each pristine MBG.

**Table 2.** Single-pulse acquired ^29^Si nuclear magnetic resonance data. Deconvolution results for the NMR spectra recorded by single pulses. Each peak component is characterized by a chemical shift (*δ*; accurate within ±0.25 ppm), relative population (‘fraction’; uncertainty ±2 percentage units) and full width at half maximum (FWHM; accuracy ±0.3 ppm).

	*Q*^4^			*Q*^3^_H_			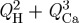 ^a^			*Q*^2^_Ca_			*Q*^1^_Ca_		
sample	−*δ* (ppm)	FWHM (ppm)	fraction (%)	−*δ* (ppm)	FWHM (ppm)	fraction (%)	−*δ* (ppm)	FWHM (ppm)	fraction (%)	−*δ* (ppm)	FWHM (ppm)	fraction (%)	−*δ* (ppm)	FWHM (ppm)	fraction (%)
S90	109.8	10.7	67.4	100.3	9.5	23.0	91.0	10.0	5.9	82.5	8.9	2.5	74.0	10.0	1.2
S90-0.5h	110.2	9.8	67.5	100.4	8.3	23.1	91.2	10.0	7.0	82.5	7.6	1.4	75.0	6.9	1.0
S90-4h	110.2	9.5	69.5	100.1	8.2	24.8	91.1	6.9	4.6	83.3	5.0	1.1			
S90-1d	110.7	9.3	72.9	100.7	7.9	24.1	91.5	7.5	3.0						
S90-3d	110.8	9.1	73.2	100.9	8.0	24.4	91.3	6.0	2.4						
S90-7d	110.8	9.0	74.2	101.0	7.9	23.5	91.5	7.9	2.3						
S85	109.7	11.2	75.1	100.2	8.5	17.8	92.2	12.0	7.1						
S85-0.5h	110.5	10.2	75.1	100.8	7.1	17.9	93.9	12.0	7.0						
S85-1h	109.6	9.7	75.3	100.0	7.4	21.1	92.0	8.0	3.6						
S85-4h	110.9	9.3	72.0	101.4	7.8	24.4	93.0	7.9	3.6						
S85-1d	110.9	9.1	72.8	101.3	7.8	23.8	91.9	7.3	3.4						
S85-3d	111.1	9.0	72.6	101.5	7.3	23.8	93.0	8.8	3.6						
S85-7d	111.0	9.1	76.0	101.3	7.3	21.1	93.0	8.8	2.9						
S58	108.8	11.8	44.6	99.3	8.3	18.3	91.5	10.5	18.2	83.0	9.3	13.5	76.0	8.4	5.4
S58-7d	110.3	10.5	45.0	99.4	9.0	24.7	91.4	10.5	17.4	83.0	10.0	10.4	74.2	9.5	2.5

^a^For the series of S90 and S85 samples, this NMR peak stems exclusively from *Q*^2^_H_ units, whereas it carries contributions from *bothQ*^2^_H_ and *Q*^3^_Ca_ tetrahedra for the S58-deriving samples.

According to predictions (**A**)–(**C**) in §3*d*, the average silicate network connectivity should *increase* along the series S58<S90<S85. This is indeed witnessed by the steadily decreased signal intensity in the higher ppm region of the respective NMR spectra in figures 3 and 4. We note that suggestion (**B**) is confirmed by the absence of significant signal intensity in the spectral range higher than −85 ppm observed from the S85 MBG, which is consistent with very low contributions from Ca^2+^-associated tetrahedra in its silicate network. The S90 sample, on the other hand, reveals weak but significant NMR responses in this spectral region, whereas they are substantial in those recorded from S58. As expected, the relative populations of *Q*^4^ tetrahedra decrease monotonically along the series S85>S90>S58, amounting to 75%, 67% and 45%, respectively (table 2).

Interestingly, the relative amount of *Q*^3^_H_ species remains at around 20% at all three MBG surfaces. While the population of Si–OH groups displays some dependency on the Ca content at the surface (through its accompanying water association), it is primarily reflected by the total surface area of the sample; the latter is very similar for the S90 and S85 MBGs, but lower for S58 [22–24]. The *Q*^2^_H_ population is also similar at the S85 and S90 MBG surfaces, as expected, whereas a two to three times larger NMR peak intensity is observed around −92 ppm from the S58 sample. This reflects the presence of significant contributions from *Q*^3^_Ca_ units in this MBG (consistent with its high Ca content), whose NMR signals overlap with those of the *Q*^2^_H_ tetrahedra (see §3*a*). This verifies prediction (**C**).

The quantitative predictions from equation (3.2) were verified by calculating the NMR-derived average number of BO atoms (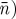 at the silicate tetrahedra of each MBG sample. We stress that equation (3.2) only accounts for the network defragmentation stemming from Ca^2+^ modifiers: hence, the set of experimental fractions {*x*_*n*_} of the *Q*^4^ and *Q*^*n*^_Ca_ tetrahedra listed in table 2 were renormalized to a unity sum. We assumed that all NMR signals around −92 ppm stem solely from 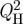 units in the S90 and S85 samples, whereas the corresponding signal fraction (18%; table 2) is shared equally between *Q*^2^_H_ and *Q*^3^_Ca_ species for the case of S58. Then, the expression 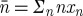 estimated the average number of BO atoms to 4.00, 3.88 and 3.28 in the silicate networks of S85, S90 and S58, respectively. These values may be contrasted with the respective predictions of 3.90, 3.80 and 3.13 from equation (3.2). The excellent agreement (within 5% throughout) is gratifying when considering the uncertainties involved in the analysed sample compositions, the NMR-derived fractional populations, as well as the assumptions leading to equation (3.2).

#### (ii) ^1^H→^29^Si cross polarization: the mesoporous bioactive glass surface

**Figure 5.**
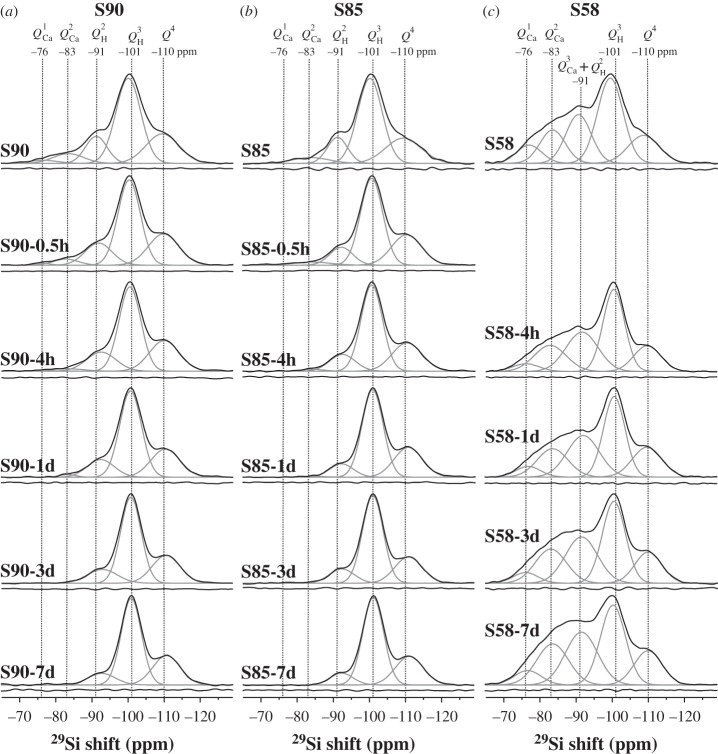
^1^H→^29^Si CPMAS spectra (black traces) from pristine (top row) and SBF-exposed specimens of the (*a*) S90, (*b*) S85 and (*c*) S58 MBG systems. Other labels are as in figure 3.

The surface specificity of the ^1^H→^29^Si CP acquisitions becomes very evident when contrasting the NMR spectra depicted in figure 5 with those recorded by single pulses (figures 3 and 4). All the former reveal weak signals from *Q*^4^ tetrahedra and are dominated by the ^29^Si resonance from the 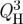 silanol groups, most notably for the spectra recorded from the S90 and S85 samples, where the *Q*^*n*^_H_ groups constitute the *majority* of the surface silicate speciation and the minute amounts of Ca^2+^ produce insignificant surface-network modifications. The NMR parameters and fractional populations determined by spectral deconvolution of the CPMAS spectra are listed in table 3: they overall accord with the inferences from the *Q*^*n*^ speciations representative of the entire silicate reservoir (table 2), in which we highlight the following:

— The S85 and S90 surfaces manifest a very similar *Q*^3^_H_ population (approx. 53%), whereas that associated with S58 is slightly lower (39%).
— Essentially identical *Q*^2^_H_ populations (approx. 14%) are observed from the S85 and S90 MBGs, reflected by the ^29^Si resonance around −91 ppm in figure 5. This peak intensity is markedly larger from the Ca-rich S58 sample, translating into a net relative population of 22% (table 3) from its two *Q*^3^_Ca_ and *Q*^2^_H_ contributions. However, their net signal *enhancement* (compared with the cases of S90 and S85) is less pronounced in these CP-acquired NMR spectra than in their directly excited counterparts (table 2). This is attributed to the distinct experimental responses from the *Q*^2^_H_ and *Q*^3^_Ca_ species: despite the signal contribution from the 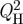 tetrahedra being emphasized in the ^1^H→^29^Si CP-acquired NMR spectrum from the Ca-rich S58 sample, a significant fraction of its total *Q*^3^_Ca_ reservoir is present *inside* the pore wall, whose ^29^Si NMR signal remains undetected by CP. The combination of these counteracting effects accounts for the almost equal (approx. 20%) relative peak areas observed in the NMR spectra recorded by using either CP or single pulses.
— As opposed to the case of S58, where a significant, but not predominant, portion of all Ca-associated *Q*^*n*^_Ca_ units is located at/near the pore-wall surface, essentially *all* such units in the S90 and S85 structures constitute surface species. This is evidenced by comparing the populations of the *Q*^2^_Ca_ and *Q*^1^_Ca_ tetrahedra in tables 2 and 3: while their contributions are insignificant/absent in the directly excited NMR spectra, they are markedly enhanced in those recorded by CP.

**Table 3.** ^1^H→^29^Si CPMAS NMR data. Results of deconvoluting the CPMAS NMR spectra. Notation and experimental uncertainties are as in table 2.

	*Q*^4^			*Q*^3^_H_			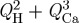			*Q*^2^_Ca_			*Q*^1^_Ca_		
sample	−*δ* (ppm)	FWHM (ppm)	fraction (%)	−*δ* (ppm)	FWHM (ppm)	fraction (%)	−*δ* (ppm)	FWHM (ppm)	fraction (%)	−*δ* (ppm)	FWHM (ppm)	fraction (%)	−*δ* (ppm)	FWHM (ppm)	fraction (%)
S90	109.5	10.7	24.0	100.1	8.1	52.1	91.2	7.0	14.3	83.5	10.0	7.3	76.3	9.8	2.3
S90-0.5h	109.9	10.0	26.3	100.3	7.3	52.3	91.8	8.8	16.4	82.7	8.0	4.1	76.0	5.1	0.9
S90-4h	110.0	9.7	26.8	100.5	7.1	53.8	92.6	9.6	16.5	83.0	11.0	2.4	76.0	12.0	0.5
S90-1d	110.3	9.4	25.4	100.7	7.1	58.4	92.4	8.8	14.9	83.3	5.1	1.3			
S90-3d	110.6	9.1	26.4	100.8	6.7	59.6	92.9	9.7	14.0						
S90-7d	110.7	8.9	28.1	100.9	6.5	60.8	92.7	8.9	11.1						
S85	109.4	11.7	24.5	100.2	7.9	55.5	91.1	6.8	14.9	83.0	11.6	5.1			
S85-0.5h	110.3	9.8	27.3	100.6	7.2	56.3	92.2	8.1	13.5	82.0	12.0	2.9			
S85-1h	110.5	9.7	29.9	100.7	7.1	55.9	92.4	8.1	12.5	83.0	10.3	1.7			
S85-4h	110.4	9.8	26.9	100.7	6.9	57.6	92.6	8.9	14.5	83.0	5.0	0.8			
S85-1d	110.8	9.2	28.0	100.9	6.8	60.6	92.3	8.0	11.0	82.0	5.1	0.4			
S85-3d	110.9	8.9	25.0	101.0	6.7	62.7	92.4	8.1	12.3						
S85-7d	111.1	9.1	27.6	101.0	6.8	63.0	92.3	7.5	9.4						
S58	108.7	10.1	15.6	99.5	8.3	39.0	90.7	8.2	22.1	83.5	8.2	15.1	77.0	8.2	8.2
S58-1h	109.3	9.2	15.6	100.2	7.0	34.5	91.5	10.7	27.9	83.0	10.5	16.2	77.0	9.6	5.8
S58-4h	109.7	8.8	14.6	100.4	7.0	36.9	91.7	10.5	26.6	82.8	10.5	17.6	76.1	8.7	4.3
S58-1d	109.6	9.5	17.2	100.6	7.0	34.1	92.0	10.5	26.3	83.5	10.0	17.2	76.7	8.1	5.2
S58-3d	110.1	8.7	15.1	100.5	7.5	34.1	91.5	10.5	26.9	83.0	10.1	19.2	76.0	8.1	4.7
S58-7d	109.9	9.0	15.0	100.2	8.2	31.5	91.5	10.5	26.8	83.5	10.4	20.6	76.6	8.8	6.1

### (b) Simulated body fluid-exposed samples

#### (i) Reactions at the S90 and S85 surfaces

**Figure 6.**
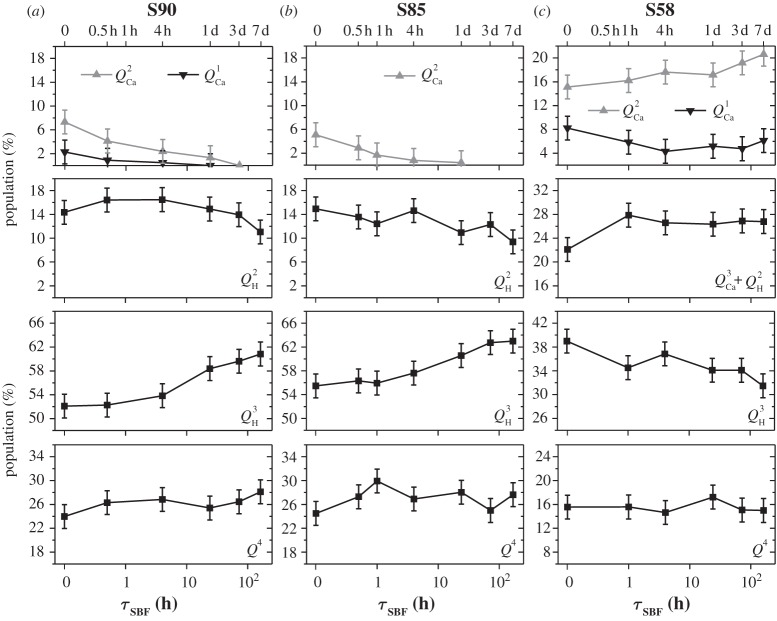
Plots of the as-indicated *surface* silicate speciations (obtained from the CPMAS NMR spectra of figure 5) against the SBF exposure interval (*τ*_SBF_). The results correspond to those of the (*a*) S90, (*b*) S85 and (*c*) S58 MBGs. Note the use of a logarithmic time-scale (bottom) and that the vertical scale of all plots employs the same span of values, despite the limiting values varying.

We now turn to the ^29^Si NMR results from the series of SBF-soaked S90 and S85 samples (tables 2 and 3) and focus mainly on the CPMAS-derived silicate surface speciations plotted in figure 6.

Both MBG series display the same trends during one week of SBF soaking. Three primary processes may be identified, as follows. (i) Dissolution of surface-associated Ca^2+^ions, according to
4.1
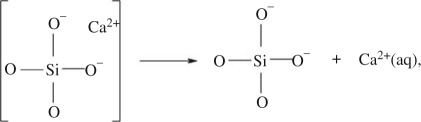
leads to a nearly complete removal of *Q*^2^_Ca_ (and for S90 also *Q*^1^_Ca_) tetrahedra over the first 4 h of SBF soaking, which emphasizes their preferential *surface association*. (ii) The S90 MBG surface reveals a slightly increased *Q*^2^_H_ population emerging over the first 24 h. This is naturally attributed to the Ca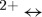 H^+^ exchange, leading to the transformation of *Q*^2^_Ca_ tetrahedra into *Q*^2^_H_ (i.e. geminal silanol moieties),
4.2
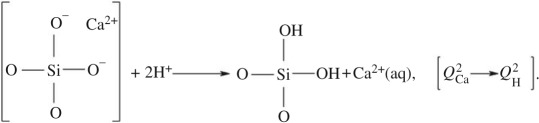
(iii) However, the *aggregate* surface modification after one week of SBF exposure constitutes a network *repolymerization*, verified by a steadily increased *Q*^3^_H_ population and a concurrent decrease in that of *Q*^2^_H_, which is observed from both MBG series over the full week of SBF immersion (figure 6). This observation may be rationalized from *Q*^3^_H_ tetrahedra forming by the condensation of two neighbouring *Q*^2^_H_ units [14], according to
4.3
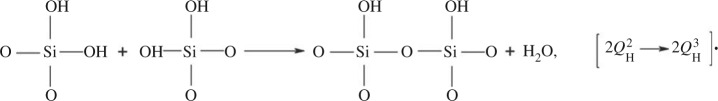
Both S85 and S90 MBG surfaces also hint at slightly increased *Q*^4^ populations after one week of SBF immersion. For the case S90, the elevated relative amount of *Q*^4^ tetrahedra is also supported by the NMR results obtained from single-pulse excitation (table 2 and figure 3).

In summary, the surface reactions (i)–(iii) imply a net sequence of conversions according to 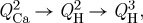 i.e.
4.4
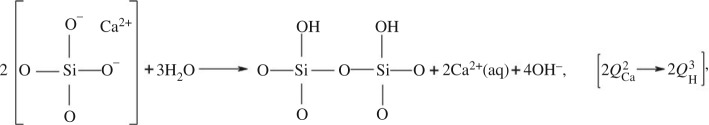
which amounts to a slight overall repolymerization of the silicate surface. We stress that equations (4.1)–(4.4) represent schematic reactions: all these processes are dynamic, mutually coupled and involve *all* distinct *Q*^4^, *Q*^*n*^_H_ and *Q*^*n*^_Ca_ species. Table 3 and figure 6 verify that, for both the S90 and S85 MBGs, the net *decrease* of the *Q*^2^_Ca_, *Q*^1^_Ca_ and *Q*^2^_H_ populations matches well with the simultaneous *increase* of the 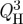 species.

**Figure 7.**
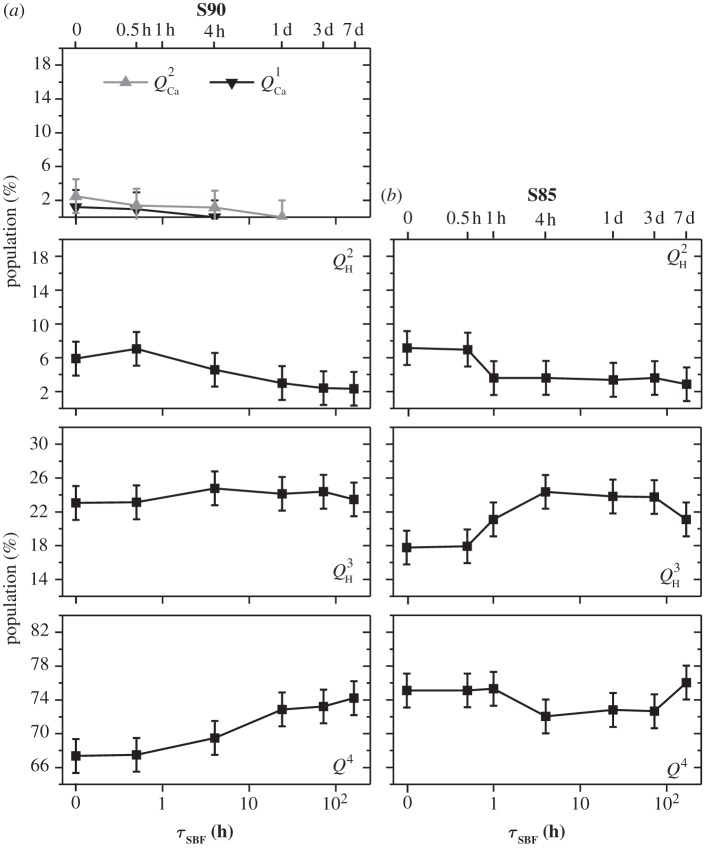
Silicate speciations quantitatively reflecting each entire sample, as obtained from the directly excited ^29^Si NMR spectra (displayed in figure 3) from (*a*) S90 and (*b*) S85 and plotted against the SBF-soaking interval.

We have focused on the MBG surface reactions during SBF treatment, as inferred from the silicate speciations derived from ^1^H→^29^Si CPMAS NMR. However, the gross trends of (i) depleted *Q*^*n*^_Ca_ species, (ii) the slight decrease in the *Q*^2^_H_ populations, and (iii) the simultaneous increase in the *Q*^3^_H_ silanols may also be verified from the single-pulse NMR results plotted in figure 7: while the trends become less transparent owing to the dominating *Q*^4^ populations that obscure the changes in the contributions from surface-associated units, the *quantitative* SiO_4_ speciations presented in table 2 account for the net modifications across the *entire* sample, thereby further supporting the inferences made from CPMAS NMR.

#### (ii) S58 surface reactions

The conclusions drawn from the NMR results of the Ca-rich S58 sample confirm some of the trends observed for the S90 and S85 series, but differ in several aspects: generally, this specimen behaves closer to MPBGs in terms of network defragmentation. Relative to the S58 sample prior to SBF treatment, the directly excited ^29^Si NMR spectrum from S58-7d (figure 4) manifests a lower signal intensity in the chemical shift region higher than −90 ppm, which verifies a net release of Ca^2+^ ions.

Focusing on the various silicate populations obtained by ^1^H→^29^Si CPMAS, listed in table 3 and plotted in figure 6, a decrease in the amount of *Q*^1^_Ca_ units is observed for prolonged SBF-soaking intervals. However, contrary to the case of the S90 and S85 surfaces, there are no signs of a total surface depletion of Ca^2+^ ions. Rather, the *Q*^2^_Ca_ population merely *increases* slightly from approximately 15% to approximately 21%. Owing to the complexities arising from the six co-existing *Q*^4^, *Q*^3^_H_, *Q*^2^_H_, *Q*^3^_Ca_, *Q*^2^_Ca_ and *Q*^1^_Ca_ structural units at this very complex MBG surface, the spectral deconvolutions and peak assignments become less certain. Further complications arise as, for instance, the *Q*^1^ species are here pragmatically attributed solely to *Q*^1^_Ca_, but may involve simultaneous charge balance from Ca^2+^ and protons, i.e. SiO(O^−^)_2_OH and/or SiO(O^−^)(OH)_2_ species associated with one and 1/2 Ca^2+^ ion, respectively. Likewise, the NMR signal intensity around −83 ppm, thought to stem entirely from *Q*^2^_Ca_ units, may receive contributions from SiO_2_(O^−^)OH moieties. Nevertheless, the NMR spectra from *all* S58-*τ*_SBF_ samples reveal a substantial intensity in the shift region higher than −90 ppm, strongly suggesting significant remains of surface-associated Ca^2+^ ions.

The net surface reactions occurring over one week of SBF exposure reflect a network *depolymerization*, where *Q*^3^_H_ silanols are replaced by lower connectivity *Q*^2^_H_ and *Q*^2^_Ca_ units (and possibly also tetrahedra receiving charge balance by *both* Ca^2+^ and H^+^), whereas the *Q*^4^, population stays essentially constant throughout. The 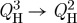 conversions occurring during the first hour of SBF immersion (figure 6) accord with the expectations of the second step of the HM (figure 1), which, incidentally, is *not*observed for the other MBGs. However, the *Q*^3^_H_ population steadily decreases over one week, whereas those of *Q*^3^_Ca_ and *Q*^2^_H_ remain constant after the first hour: hence, between 1 h and one week of S58 soaking, the net reactions involve transformation of *Q*^3^_H_ into *Q*^2^_Ca_ or ‘mixed’ SiO_2_(O^−^)OH tetrahedra.

These results indicate a closer behaviour of the S58 MBG to that typically observed for MPBGs *in vitro*; they may also stem from the high MBG concentration in the SBF solution, leading to abnormal MBG surface reactions and retarded HCA formation from this Ca-rich composition [40]. While S85 evidenced HCA formation within 24 h of SBF soaking, neither powder XRD nor ^31^P NMR revealed any traces of HCA from the present S58 sample [40].

## 5. Mesoporous bioactive glass surface reactions and the Hench mechanism

Overall, the surface reactions observed from the S90 and S85 MBGs accord well with the predictions of the HM (figure 1). Equations (4.1) and (4.2) reflect the initial step of Ca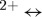 H^+^ cation exchange. The subsequent silicate network defragmentation of the second HM stage is evident only from the S58 sample. Nevertheless, MBG surfaces already *inherently* comprise low-connectivity *Q*^*n*^_H_ tetrahedra (*n*=2, 3), which markedly accelerate the surface reactions when compared with the analogous processes associated with MPBGs [14]. The third HM step, involving network repolymerization at the surface, is witnessed by the increased amounts of *Q*^3^_H_ (and to a minor degree *Q*^4^) units by condensation of *Q*^2^_H_ tetrahedra (equation (4.3)). The aggregate effects of the first three HM stages are summarized (schematically) as equation (4.4).

The HM was proposed to account for the formation of HCA from MPBGs that comprise significantly larger amounts of network modifiers than the present MBGs, thereby translating into open structures built primarily by *Q*^2^ units (and to a lesser extent *Q*^3^). Therefore, both the HM stages 2 and 3 naturally become more pronounced for such structures. For instance, ^29^Si MAS NMR unambiguously evidenced the formation of *Q*^4^ tetrahedra after prolonged SBF soaking of MPBGs [11,47], which was readily detectable as the initial structures do not involve such building blocks. For MPBGs, the first three stages of the HM lead to the formation of a ‘silica-gel’ layer and accompanying *increased surface area* of the material, as observed/discussed intensively in the literature [6–11]. While formally this feature also applies to the *Ca-poor* S90 and S85 MBGs, their surface alterations become less pronounced as the Ca^2+^ leaching is low. Indeed, the MBG surface is already ‘gel-like’, as it is rich in both silanols and physisorbed water molecules. In the study of Gunawidjaja *et al*. [14], we proposed that the MBG surface reactions generally accelerate, and may even partially circumvent, the three initial stages of the HM. This is one reason for the enhanced bioactivity observed from MBGs [13,14,20–24], although the CaP clusters also play a significant role in the enhanced rate of HCA formation [14,23].

## 6. ^1^H nuclear magnetic resonance

**Figure 8.**
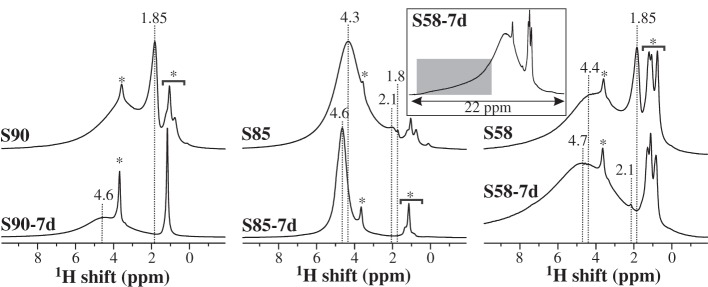
^1^H MAS NMR spectra recorded by Hahn spin–echoes [27] from each MBG before (top row) and after (bottom row) SBF exposure for one week. The signals around 4.5 ppm and 2 ppm stem from physisorbed water molecules and isolated silanols, respectively, whereas the sharp peaks marked by asterisks (approx. 3.6 ppm and less than 1.3 ppm) derive from minor residues of organic templating molecules. The inset spectrum is a horizontal expansion of that from S58-7d, where resonances from strongly hydrogen-bonded silanols are indicated by the grey rectangle. Note that the NMR spectra are normalized to the same maximum amplitude and do not reflect absolute intensities.

^1^H NMR is useful for identifying the various proton environments present at the MBG surface. Here, we merely briefly review the most important aspects of the ^1^H NMR spectra recorded from our present samples (figure 8), and refer to Leonova *et al*. [23] for a detailed account with further motivations for the peak assignments.

Disregarding the narrow NMR signals marked by asterisks in figure 8, which originate from minor residual organic precursor/templating molecules, three main groups of surface protons may be identified, as follows. (i) Those of physisorbed water molecules and SiOH moieties resonating between 4 ppm and 5 ppm [32–35,48]. (ii) Protons of ‘isolated’ silanols not experiencing hydrogen bonding [32–35] that reveal two relatively sharp NMR peaks at 1.85 ppm and 2.1 ppm. While the former signal is usually more prominent, e.g. in the spectra from S90 and S58 in figure 8, the latter is visible in those of S85 and S58-7d (also see [23]). (iii) Strongly hydrogen-bonded silanols that produce broad ^1^H resonances in the spectral region higher than 6 ppm [32,33,35,48]. This surface feature is prominent only in the NMR spectra of the S58-derived samples, as indicated by the inset shown in figure 8. The significant calcium content of the S58 MBG makes its surface hydrophilic, thereby leading to an enhanced water association [23]; this is evidenced by the much stronger and broader NMR signal around 5 ppm (from water molecules) present in the spectrum from S58-7d relative to those derived from S90 and S85 (figure 8).

The main distinction among these groups of O^1^**H** environments is their extent of participation in hydrogen bonding (in turn dictated by the amount of physisorbed water molecules), which primarily determines the precise ^1^H NMR peak position (around 4–5 ppm) originating from SiOH/H_2_O environments: the stronger the hydrogen bonding, the higher the chemical shift [31,48,49]. This is witnessed by the displacement of the NMR signal to higher shifts, which is consistently observed after SBF treatment of each MBG sample (figure 8). The peak deshielding (and accompanying increased water content at the surface) is also coincident with the disappearance of NMR signals from ‘isolated’ silanols: naturally, the latter exist only at ‘dry’ MBG surfaces [23].

## 7. Summary

The key experimental tools of this work consisted of solid-state ^29^Si and ^1^H NMR [30,31], whose utilities were reviewed and illustrated for quantitatively monitoring the silicate speciation of both the MBG pore-wall interior and its surface for three MBG specimens of different Ca, Si and P contents. Excellent agreement was observed between the NMR-estimated average number of BO atoms per silicon tetrahedron, and that predicted by a split-network analysis [42,43]. We also discussed the various proton environments of silanols and surface-adsorbed water molecules revealed by ^1^H NMR.

For each of the three MBG systems, we presented experimental results unveiling the silicate surface alterations following SBF exposure for intervals between 30 min and one week. For two MBGs associated with low Ca contents, either phosphorus bearing (S85) or devoid of P (S90), ^29^Si CPMAS evidenced both release of Ca^2+^ ions from the surface and an overall condensation of its silicate network on prolonged SBF immersion, which suggested the following schematic sequence of tetrahedral unit conversions: 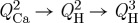 (equation (4.4)). The surface reactions observed herein were contrasted with those proposed in the context of traditional melt-prepared BGs by Hench [1,6] (figure 1). The S90 and S85 MBGs displayed similar *in vitro* behaviour, whereas the Ca-richer S58 specimen was observed to react more similarly to MPBGs. Yet, this feature of the S58 MBG composition is believed to partially originate from the relatively high MBG concentrations employed in our *in vitro* testing, which for the case of the Ca-rich S58 sample may lead to abnormal surface reactions [40]. Our future work will aim at elucidating this further by comparison with NMR results obtained from conditions of lower MBG loading per SBF volume, as well as investigating the similarities and distinctions between the mechanisms leading to HCA formation *in vitro* from MBGs when compared with MPBGs.

The present contribution focused on examining the reactions involving the *silicate surface* species of relevance for the three initial steps of the HM (figure 1). Here, ^29^Si CPMAS NMR is the most natural and informative probe, whereas ^31^P and ^1^H NMR are the methods of choice to study the HCA formation itself (i.e. stages 4 and 5 of the HM); we refer to complementary studies [14,40,41] for results that monitor the evolution of the biomimetic phosphate layer forming in SBF solutions.
